# TBK1 promotes thyroid cancer progress by activating the PI3K/Akt/mTOR signaling pathway

**DOI:** 10.1002/iid3.796

**Published:** 2023-03-14

**Authors:** Qiuli Jiang, Yingying Guan, Jingmei Zheng, Huadong Lu

**Affiliations:** ^1^ Department of Pathology, Xiamen Branch, Zhongshan Hospital Fudan University Xiamen Fujian P. R. China

**Keywords:** MAZ, migration, proliferation, TBK1, thyroid cancer

## Abstract

**Introduction:**

Thyroid cancer has received increasing attention; however, its detailed pathogenesis and pathological processes remain unclear. We investigated the role of TANK‐binding kinase 1 (TBK1) in the progression of thyroid cancer.

**Methods:**

The expression of TBK1 in thyroid cancer and normal control tissues was analyzed using real‐time quantitative polymerase chain reaction. The function of TBK1 on thyroid cancer cells was detected using MTT, colony formation, wound healing, and Transwell assays. The xenograft assay was carried out to check on the role of TBK1 in thyroid cancer.

**Results:**

TBK1 was highly expressed in thyroid tumors. High expression of TBK1 raised viability, proliferation, migration, and invasion of thyroid cancer cells. Gene set enrichment analysis revealed that TBK1 activated the phosphatidylinositol‐3‐kinase/protein kinase B/mammalian target of rapamycin pathway. In addition, Myc‐associated zinc finger protein (MAZ) was overexpressed in thyroid cancer and transcriptionally activated BK1. MAZ silence reversed the effects of TBK1 overexpression on thyroid cancer progression. Cotransfection with MAZ small‐interfering RNA(siRNA) and TBK1 siRNA did not strengthen the inhibitory effect of TBK1 silencing on the thyroid cancer cells. The xenograft tumor assay showed that TBK1 short hairpinRNA inhibited tumor growth.

**Conclusion:**

MAZ silencing inhibited tumor progress of thyroid cancer cells, whereas this inhibitory effect was reversed by TBK1 overexpression.

## Graphical abstract

Myc‐associated zinc finger protein (MAZ) silence significantly inhibited cell viability, proliferation, migration, and invasion of thyroid cancer cells, whereas the inhibitory effect was reversed by TANK‐binding kinase 1 (TBK1) overexpression.

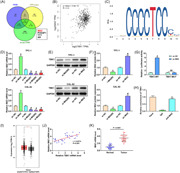

## 1 INTRODUCTION

Thyroid cancer, one of the high‐incidence cancers in many countries, has attracted increasing attention.^1^, ^2^ Although most patients with thyroid cancer have a relatively good prognosis after surgery, widespread lymph node metastasis and local recurrence persist. Several studies on thyroid cancer are mainly related to the expression of sphingosine kinase 2,^3^, ^4^ microRNA (miR)‐155,^5^ miR‐146b‐5p,^6^ miR‐3619‐3p,^7^ lysophosphatidic acid receptor 5, SIX1, E3 ligases,^8^ and KEAP1/CUL3/RBX1E3–ubiquitin ligase complex.^9^ In addition, the pathogenesis of thyroid cancer is related to many signaling pathways, including nuclear factor‐κB (NF‐κB),^10^ Wnt/β‐catenin, phosphatidylinositol‐3‐kinase/protein kinase B (PI3K/Akt),^11^ and signal transducer and activator of transcription 3 signaling pathway.^12^ Although several studies highlight the genes and signaling pathways associated with thyroid cancer, more research is needed on the mechanisms underlying thyroid cancer progression.

TANK‐binding kinase 1 (TBK1) regulates innate immune responses by coordinating the activation of key transcription factors, such as interferon regulatory factor 3 and the NF‐κB pathway.^13^, ^14^ TBK1, a gene screened by yeast two‐hybrid experiments, is expressed in most cell types.^15^ TBK1 has become a prospective therapeutic target and plays an increasingly important role in autoimmune diseases, metabolic diseases, and cancers.^16^, ^17^ Inhibition of TBK1 resulted in decreased cell viability and proliferation of lung cancer cells.^18^ Although TBK1 is considered a target of cancer, its related mechanisms remain unclear.

In recent years, the association between TBK1 and tumors has been increasingly studied and involves a variety of cancer types. TBK1 acts on and inhibits the function of the mammalian target of rapamycin protein (mTOR), arresting the cell cycle of prostate cancer and improving the resistance of PCa stem cells to chemotherapy.^19^ In breast cancer, TBK1 is overexpressed in estrogen receptor α (ERα)‐positive breast cancer. Because the expression of TBK1 is consistent with that of ERα, TBK1 may become an important prognostic marker of breast cancer subtypes.^20^ TBK1 could directly act on multiple factors upstream and downstream of mTOR kinase. In lung cancer, TBK1 activated the Akt/mTOR pathway.^21^ TBK1 is related to the proliferation of oral cancer cells.^22^ These findings confirm that TBK1 is closely related to cancer and that TBK1 has an inextricable connection to the occurrence, development, apoptosis, and progression of cancer. However, whether TBK1 participates in thyroid cancer progression remains indistinct.

In the present study, we evaluated the differential expression of TBK1 in thyroid cancer and normal tissues. Subsequently, the cell functions of thyroid cancer cells were evaluated after transfection with TBK1 small‐interfering RNA(siRNA) and overexpression plasmids. In addition, we explored the signaling pathways regulated by TBK1 in thyroid cancer. Our findings provide a potential avenue for the development of therapeutic methods and targets for thyroid cancer.

## 2 METHODS

### 2.1 Patients

In this prospective study, thyroid tissues from 32 patients with thyroid cancer subjects and 20 healthy subjects were obtained from our hospital. This study was approved by the Ethics Committee of the Zhongshan Hospital. Inclusion criteria were as follows: (i) the experimental group included patients with thyroid cancer identified by pathology, and (ii) the normal group included people who were identified as nonthyroid patients. Exclusion criteria were as follows: (i) patients who had received radiotherapy and/or chemotherapy before taking specimens, and (ii) patients with serious other organ diseases.

### 2.2 Cell lines and culture

The human thyroid follicular epithelial cell line Nthy‐ori 3‐1 (EK‐Bioscience) and human thyroid cancer cells (TPC‐1, KTC‐1, 8305C, FTC‐133, and CAL‐62; Procell) were cultured in Roswell Park Memorial Institute (RPMI)‐1640 medium supplemented 10% fetal bovine serum(FBS), 100 U/mL penicillin/streptomycin, and 100 U/mL glutamine (Procell) under 5% CO_2_ and 37°C.

### 2.3 Cell transfection

siRNAs targeting TBK1 and its negative control (si‐NC), TBK1 overexpression plasmid and its negative control (pc‐NC), siRNAs targeting Myc‐associated zinc finger protein (MAZ) and its si‐NC, and MAZ overexpression plasmid and its pc‐NC were obtained from GenePharma. Thyroid cancer cells were transfected with TBK1 siRNAs and si‐NC, pc‐TBK1 and pc‐NC, MAZ siRNA and si‐NC, pc‐MAZ, and pc‐NC by using Lipofectamine 3000 for 48 h.

### 2.4 MTT assay

The viability was evaluated using an MTT Assay Kit (Beyotime).^23^ Briefly, TPC‐1 and CAL‐62 cells were cultured in a 48‐well plate (3 × 10^3^ cells/well). The MTT reagent was added when the culture reached a specific time point and incubated for 4 h, followed by the addition of dimethyl sulfoxide solution. The optical density value at 570 nm was measured by a microplate reader (Bio‐Rad Laboratories Inc.).

### 2.5 Colony formation assay

TPC‐1 and CAL‐62 cells were seeded in six‐well plates and incubated in RPMI‐1640 medium supplemented with 10% FBS at 37°C for 14 days. The cells were then fixed with 4% paraformaldehyde and dyed with 0.1% crystal violet. Finally, colonies were counted under a microscope (Olympus).

### 2.6 Wound healing assay

TPC‐1 and CAL‐62 cells were plated in six‐well plates and cultured overnight. When the cells reached 80%–90% confluence, the medium was replaced with a serum‐free medium. After 12 h of incubation, a wound was made using a pipette tip in each well. The wound healing rate was calculated at 0 and 48 h after wounding.

### 2.7 Transwell assay

The upper Transwell chamber (8 mm) was coated with Matrigel for 30 min at 37°C. Cells were added to the upper chamber and cultured in a serum‐free medium. A complete medium was added to the lower chamber. After 48 h, the invasion cells were fixed with 95% ethanol and then dyed with 0.1% crystal violet (Yeasen Biotechnology). Finally, stained cells were observed with a light microscope (Olympus).

### 2.8 Western blot

TPC‐1 and CAL‐62 cells were treated with protein lysates containing protease inhibitors. Each group maintained the same concentration of total protein, and proteins were separated using sodium dodecyl sulfate‐polyacrylamide gel electrophoresis and then transferred onto polyvinylidene difluoride membranes. The protein‐containing membranes were then treated with 5% fat‐free milk and primary antibodies (TBK1, MAZ, phospho‐PI3K [p‐PI3K], PI3K, phospho‐Akt [p‐Akt], Akt, phospho‐mTOR [p‐mTOR], and mTOR; Abcam). The membranes were then treated with a secondary antibody (Abcam) for 1 h. The proteins were imaged by an Enhanced Chemiluminescent Kit (Thermo Fisher Scientific) and analyzed by ImageJ (V1.50b; NIH).

### 2.9 Real‐time quantitative polymerase chain reaction (qRT‐PCR)

Total RNA was isolated by the TRIzol reagent (Thermo Fisher Scientific). The PrimeScript RT Reagent Kit (Takara) was used to synthesize complementary DNA. qPCR was performed using SYBR Green P0.remix Ex Taq II (Takara). The gene expression levels were calculated using the $2^{-∆∆C_{T}}$ method.^24^ Glyceraldehyde 3‐phosphate dehydrogenase (GAPDH) was used as the internal control. The primers were: TBK1, 5′‐GGAGACCCGGCTGGTATAA‐3′ (forward [F]) and 5′‐TGAACATCCACTGGACGAAGG‐3′ (reverse [R]); MAZ, 5′‐GGATCACCTCAACAGTCACGTC‐3′ (F) and 5′‐GGCACTTTCTCCTCGTGTCGTA‐3′ (R); GAPDH, 5′‐GGATTTGGTCGTATTGGGCG‐3′ (F) and 5′‐TCCCGTTCTCAGCCATGTAGT‐3′ (R).

### 2.10 Dual‐luciferase reporter assay

The target relationship between TBK1 and MAZ was verified by dual‐luciferase reporter assay.^25^ The luciferase reporter gene vectors (pGL3‐basic vector; GeneCreate) containing TBK1 mutant or wild‐type (WT) were cotransfected with pc‐MAZ or pc‐NC into 293T cells (EK‐Bioscience). After 48 h, the luciferase activity was evaluated using the Dual‐Luciferase Reporter Assay System Kit (Promega).

### 2.11 Chromatin immunoprecipitation (ChIP) assay

293T cells were fixed with 16% methanol, cross‐linked, lysed, sonicated, and then cultured with MAZ antibody (ab85725; Abcam) overnight. Beads were used to collect the protein–DNA complex. NaCl was added for cross‐linking. The enrichment of TBK1 was examined using RT‐qPCR.

### 2.12 Xenograft tumor assay

Eight 6‐week‐old female BALB/c nude mice (20 ± 2 g; Jinan Pengyue Experimental Animal Breeding Co., Ltd.) were divided into two groups (*n* = 4/group). TPC‐1 cells (5 × 10^6^ cells) transfected with shTBK1 or shNC were injected into the back of mice subcutaneously. The length and width of tumors were evaluated every 5 days. The volumes were calculated as *V* (mm^3^) = length × width^2^/2. Thirty days later, the mice were killed with pentobarbital sodium (100 mg/kg). The tumors were isolated, photographed, and weighed. The expression of TBK1 and PI3K/Akt/mTOR signaling pathway‐related proteins was detected using a western blot.

### 2.13 Statistical analysis

All data were analyzed using GraphPad Prism 7.0 and presented as mean ± SD. Comparisons between two groups were analyzed using the *t* test, and the comparisons among multiple groups were analyzed using one‐way analysis of variance. Fisher's exact test was performed to access the association between TBK1 and clinicopathological factors. *p* < .05 indicates statistical significance.

## 3 RESULTS

### 3.1 TBK1 is highly expressed in thyroid cancer tissues

**Figure 1:**
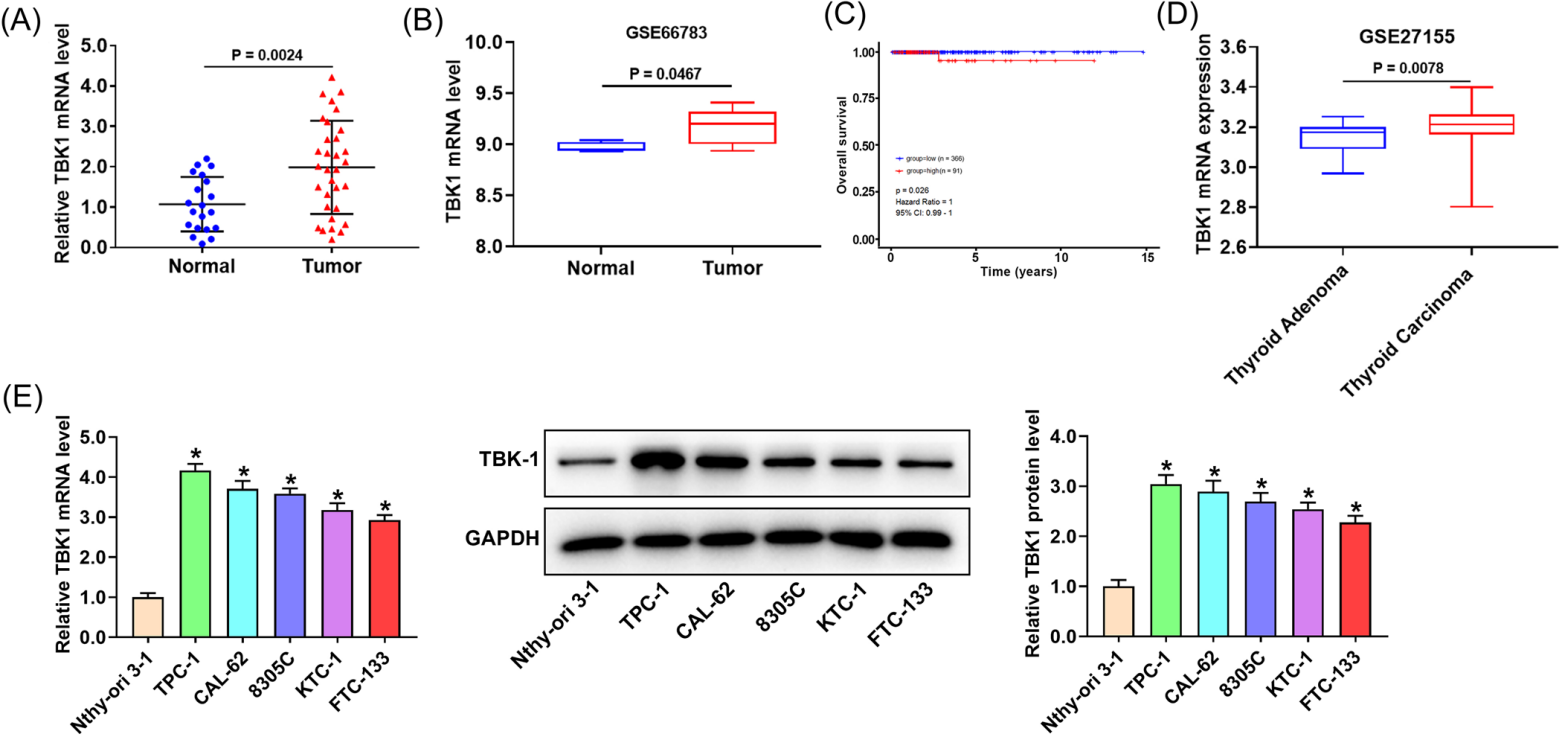
TBK1 expression is upregulated in thyroid cancer tissues. (A) TBK1 mRNA expression in thyroid tumor tissues and normal tissues. (B) TBK1 expression from the GEO data set GSE66783. (C) Overall survival analysis. (D) TBK1 expression in thyroid adenoma and thyroid carcinoma from the GEO data set GSE27155. (E) TBK1 expression in cells. Band densities were determined using ImageJ Software and quantified as the ratio to GAPDH. Take the mean value for samples from the first group on each immunoblot, and then divide the value of all groups by that mean. GAPDH, glyceraldehyde 3‐phosphate dehydrogenase; GEO, Gene Expression Omnibus; mRNA, messenger RNA;TBK1, TANK‐binding kinase 1. *p < .05.

We first analyzed TBK1 expression in thyroid tumors and normal control tissues. TBK1 was upregulated in tumor tissues (Figure 1A). Analysis of the Gene Expression Omnibus (GEO) data set GSE66783 also confirmed this finding (Figure 1B). The overall survival analysis indicated that the survival rate of thyroid cancer patients with high TBK1 expression was significantly lower than that of patients with low TBK1 expression (Figure 1C). Additionally, analysis of the GEO data set GSE27155 also confirmed that TBK1 was significantly upregulated in thyroid carcinoma compared with that in thyroid adenoma (Figure 1D). The association between TBK1 level and clinical characteristics was evaluated. The results of Table 1 revealed that TBK1 upregulation in thyroid cancer tissues was significantly associated with crucial clinicopathological factors, including T classification, N classification, histological grade, and lymph node metastasis. The TBK1 expression was higher in thyroid cancer cell lines than in thyroid follicular epithelial cell line Nthy‐ori 3‐1 (Figure 1E).

**Table 1:** The relationship between TBK1 expression level and clinical pathological characteristics in 32 patients with thyroid cancer.

Characteristics	Number	TBK1 expression		*p* Value
		Low (*N* = 16)	High (*N* = 16)	
Age, years				
≤55	20 (63%)	11	9	.716
>55	12 (37%)	5	7	
Gender				
Male	13 (41%)	8	5	.4725
Female	19 (59%)	8	11	
T classification				
T1–T2	20 (63%)	14	6	.0091
T3–T4	12 (37%)	2	10	
N classification				
N0	18 (56%)	13	5	.0113
N1	14 (44%)	3	11	
M classification				
M0	21 (66%)	12	9	.4578
M1	11 (34%)	4	7	
Histological grade				
Low	19 (59%)	13	6	.0290
Moderate/advanced	13 (41%)	3	10	
Lymph node metastasis				
No	21 (66%)	14	7	.0233
Yes	11 (34%)	2	9	

*Note*: Fisher's exact test.

Abbreviation: TBK1, TANK‐binding kinase 1.

### 3.2 TBK1 promotes cell growth of thyroid cancer cells

**Figure 2:**
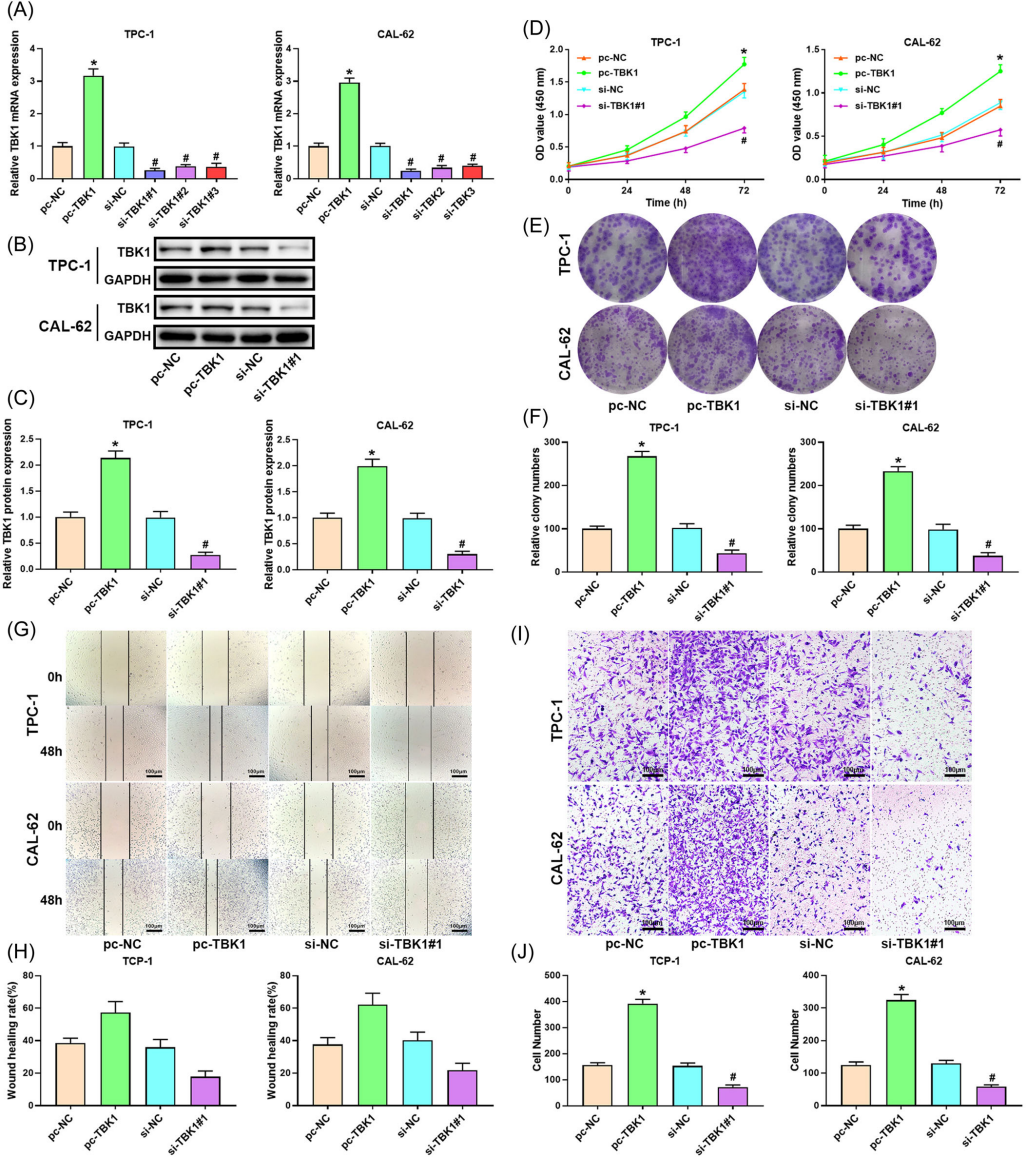
TBK1 promotes the proliferation of thyroid cancer cells. (A–C) TBK1 expression. Band densities were determined using ImageJ Software and quantified as the ratio to GAPDH. Take the mean value for samples from the first group on each immunoblot, and then divide the value of all groups by that mean. The (D) cell viability, (E, F) proliferation, (G, H) migration, and (I, J) invasion of TPC‐1 and CAL‐62 cells. Scale bar = 100 μm. *p < .05 versus pc‐NC group, ^#^ *p* < .05 versus si‐NC group. GAPDH, glyceraldehyde 3‐phosphate dehydrogenase; NC, negative control; OD, optical density; TBK1, TANK‐binding kinase 1.

To investigate the effects of TBK1 in thyroid cancer, we overexpressed and knockdown TBK1. The experimental results showed that overexpressed plasmid and siRNAs effectively regulated the expression of TBK1 (Figure 2A–C). High expression of TBK1 markedly increased the viability of thyroid cancer cells (Figure 2D). The proliferation was increased by TBK1 overexpression and decreased by TBK1 silencing (Figure 2E,F). As shown in Figure 2G,H, high levels of TBK1 significantly raised the wound healing rate of TPC‐1 and CAL‐62 cells, whereas TBK1 silencing decreased the wound healing rate of thyroid cancer cells. As shown in Figure 2I,J, the invasion cell number in TBK1‐verexpressed thyroid cancer cells was markedly raised, whereas TBK1 silencing suppressed the invasion.

### 3.3 TBK1 activates the PI3K/Akt/mTOR pathway

**Figure 3:**
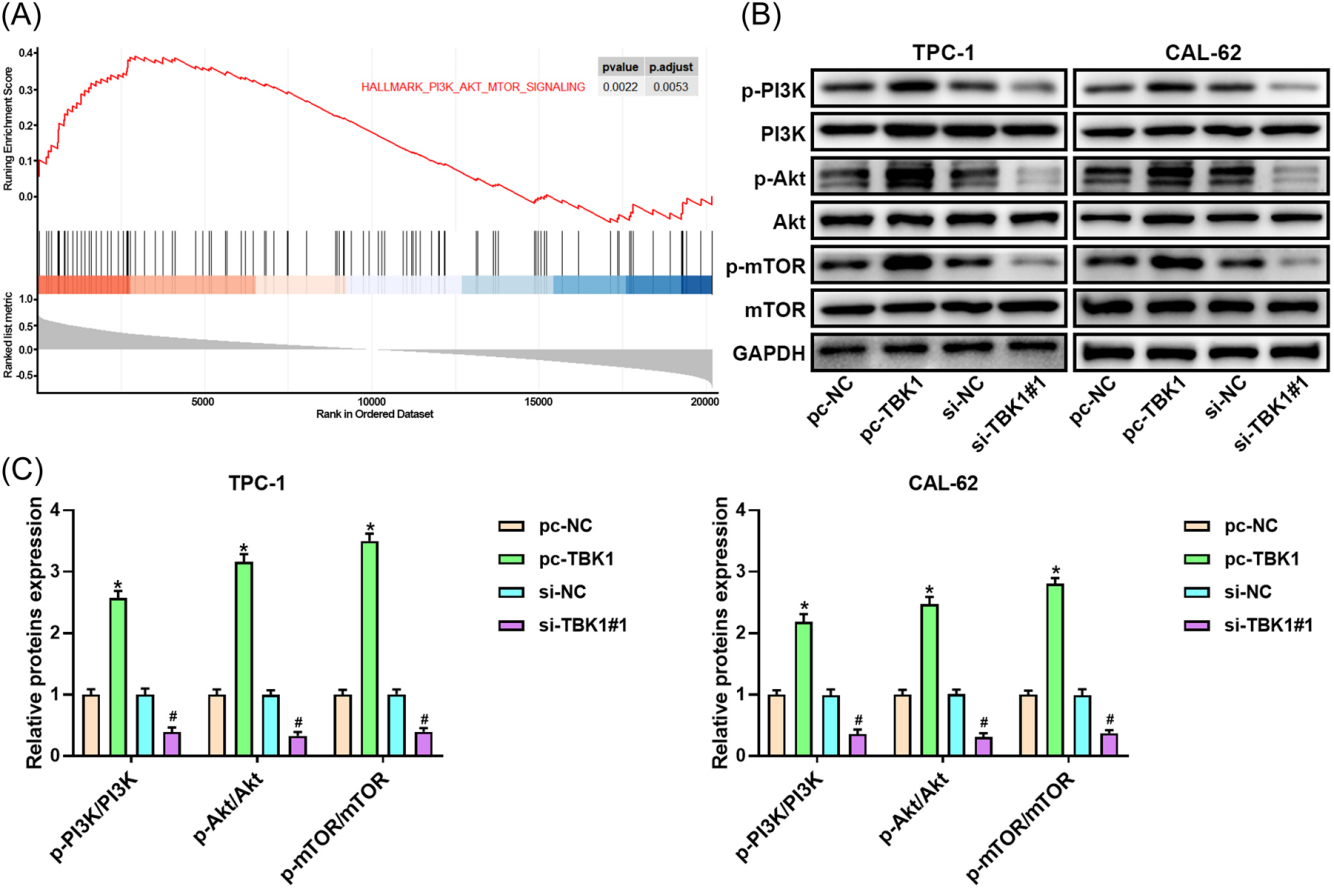
TBK1 activates PI3K/Akt/mTOR pathway in thyroid cancer. (A) GSEA analysis showed that TBK1 activates PI3K/Akt/mTOR pathway. (B, C) The expression levels of pathway‐related proteins. Band densities were determined using ImageJ Software and quantified as the ratio to GAPDH. Take the mean value for samples from the first group on each immunoblot, and then divide the value of all groups by that mean. *p < .05 versus pc‐NC group, ^#^ *p* < .05 versus si‐NC group. Akt, protein kinase B; GAPDH, glyceraldehyde 3‐phosphate dehydrogenase; GSEA, gene set enrichment analysis;mTOR, mammalian target of rapamycin protein; NC, negative control; PI3K, phosphatidylinositol‐3‐kinase; p‐Akt, phospho‐Akt; p‐mTOR, phospho‐mTOR; p‐PI3K, phospho‐PI3K; TBK1, TANK‐binding kinase 1.

Gene set enrichment analysis using the GEO data set GSE66783 was performed to explore the mechanism of TBK1 in thyroid cancer. TBK1 activated the PI3K/Akt/mTOR pathway (Figure 3A). TBK1 overexpression dramatically promoted the protein levels of p‐PI3K/PI3K, p‐Akt/Akt, and p‐mTOR/mTOR compared to that in the pc‐NC group. In contrast, the protein expression in the si‐TBK1 group was dramatically suppressed (Figure 3B,C).

### 3.4 TBK1 is transcriptionally activated by MAZ

**Figure 4:**
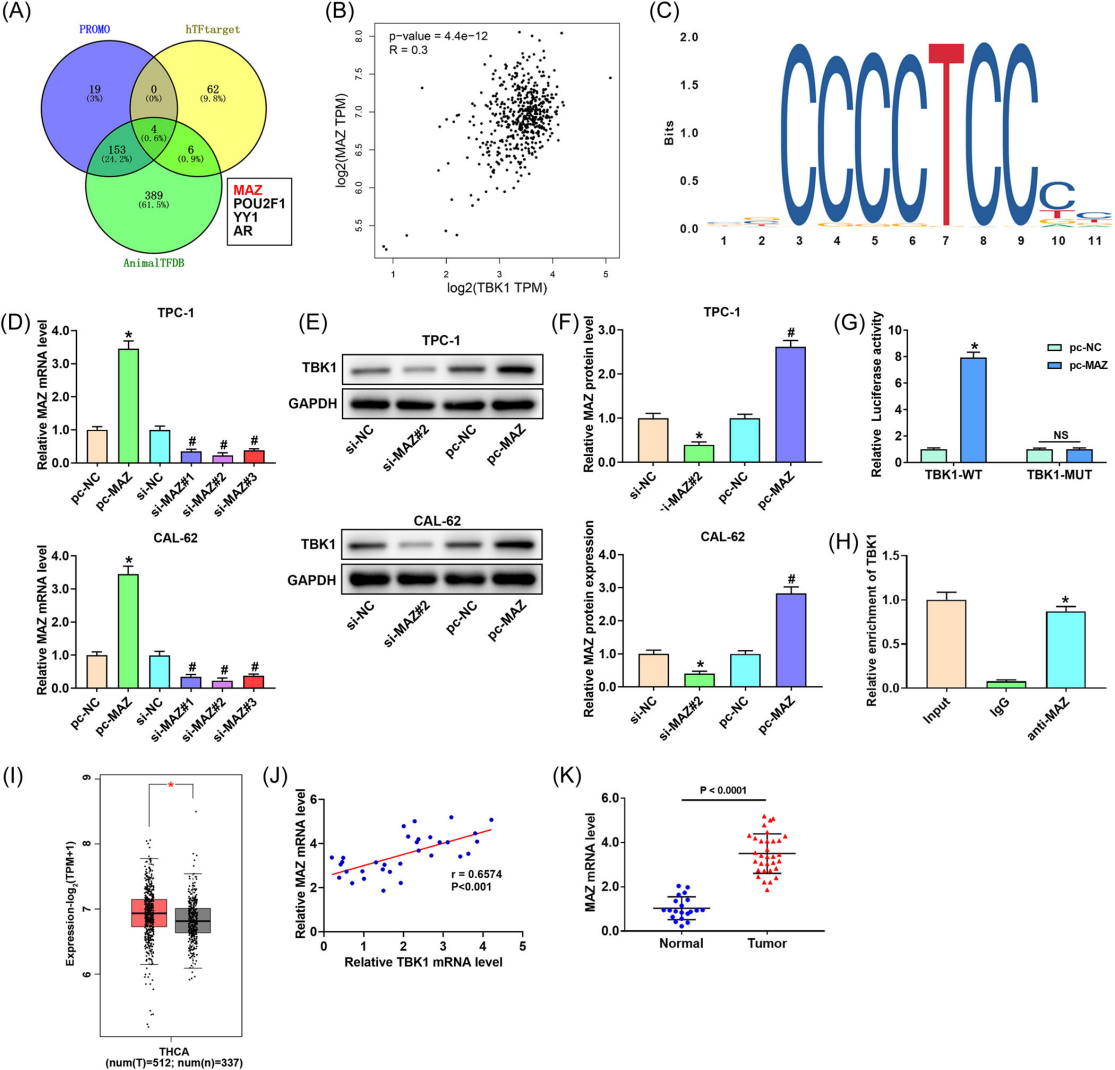
TBK1 is transcriptionally activated by MAZ. (A) The transcriptional factors that induced high expression of TBK1. (B) MAZ was positively correlated with TBK1 expression. (C) The binding motif of MAZ was predicted using the JASPAR. The (D) MAZ expression and (E, F) TBK1 expression in TPC‐1 and CAL‐62 cells. Band densities were determined using ImageJ Software and quantified as the ratio to GAPDH. Take the mean value for samples from the first group on each immunoblot, and then divide the value of all groups by that mean. *p < .05 versus pc‐NC group, ^#^ *p* < .05 versus si‐NC group. (G) Luciferase reporters assay analysis. (H) The combining capacity between MAZ and TBK1 was assessed using a chromatin immunoprecipitation assay. (I) The TBK1 expression in thyroid cancer was analyzed by the TCGA database. (J) The MAZ expression in thyroid tumors and normal tissues. (K) The correlationship between the level of TBK1 and MAZ in thyroid tumor tissues. *p < .05. GAPDH, glyceraldehyde 3‐phosphate dehydrogenase; IgG, immunoglobulin G; JASPAR, Japan Automotive Software Platform and Architecture; MAZ, Myc‐associated zinc finger protein; mRNA, messenger RNA; NC, negative control; TBK1, TANK‐binding kinase 1; TCGA, The Cancer Genome Atlas.

To identify the upstream factors inducing high expression of TBK1 in thyroid cancer, we further examined and identified four potential transcription factors using PROMO, hTFtarget, and animal Transcription Factor DataBase (Figure 4A). MAZ expression positively correlated with TBK1 expression (Figure 4B). The binding sites of MAZ and TBK1 were predicted using Japan Automotive Software Platform and Architecture (Figure 4C). MAZ overexpression significantly upregulated MAZ and TBK1 expression, whereas MAZ silencing downregulated their expression (Figure 4D–F). Additionally, MAZ overexpression increased the luciferase activity of TBK1‐WT cells (Figure 4G). ChIP assay verified the interaction between MAZ and TBK1 (Figure 4H). MAZ was highly expressed in the tumor tissues (Figure 4I,J). Additionally, there was a positive correlation between MAZ and TBK1 expression (Figure 4K).

### 3.5 MAZ silencing reverses the effect of TBK1 on thyroid cancer progression

**Figure 5:**
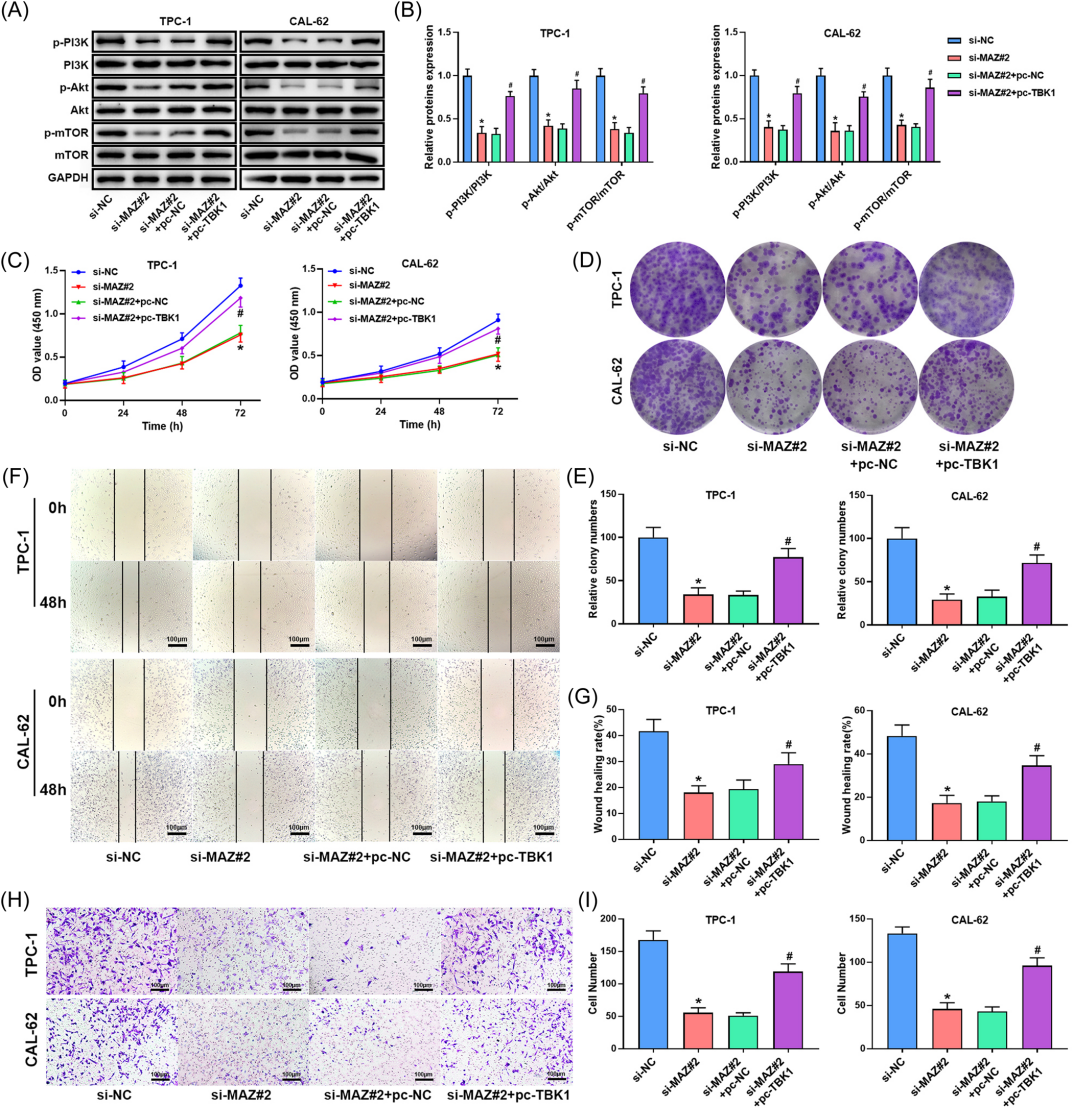
MAZ silencing reverses the effect of TBK1 on thyroid cancer progression. (A, B) The expression of pathway‐related proteins in TPC‐1 and CAL‐62 cells. Band densities were determined using ImageJ Software and quantified as the ratio to GAPDH. Take the mean value for samples from the first group on each immunoblot, and then divide the value of all groups by that mean. The (C) cell viability, (D, E) proliferation, (F, G) migration, and (H, I) invasion of TPC‐1 and CAL‐62 cells. Scale bar = 100 μm. *p < .05 versus si‐NC group, ^#^ *p* < .05 versus si‐MAZ#2 + pcNC group. Akt, protein kinase B; GAPDH, glyceraldehyde 3‐phosphate dehydrogenase; MAZ, Myc‐associated zinc finger protein; mTOR, mammalian target of rapamycin protein; NC, negative control; PI3K, phosphatidylinositol‐3‐kinase; p‐Akt, phospho‐Akt; p‐mTOR, phospho‐mTOR; p‐PI3K, phospho‐PI3K; TBK1, TANK‐binding kinase 1.

To check whether the regulatory role of TBK1 was realized by MAZ, MAZ siRNA and the TBK1 overexpression plasmid (pc‐TBK1) were cotransfected into thyroid cancer cells. MAZ silencing inhibited the PI3K Akt/mTOR pathway, whereas the inhibitory effect of MAZ silencing was reversed by TBK1 overexpression (Figure 5A,B). MAZ silencing suppressed cell viability, whereas this inhibitory effect was reversed by TBK1 overexpression (Figure 5C). Similar results were obtained for the colony formation assay. TBK1 overexpression reversed the role of MAZ silencing on cells (Figure 5D,E). MAZ silencing markedly inhibited migration and invasion, and TBK1 overexpression reversed the inhibitory effect of MAZ silencing on thyroid cancer cells (Figure 5F–I).

### 3.6 Effect of MAZ and TBK1 double knockdown on thyroid cancer cells

**Figure 6:**
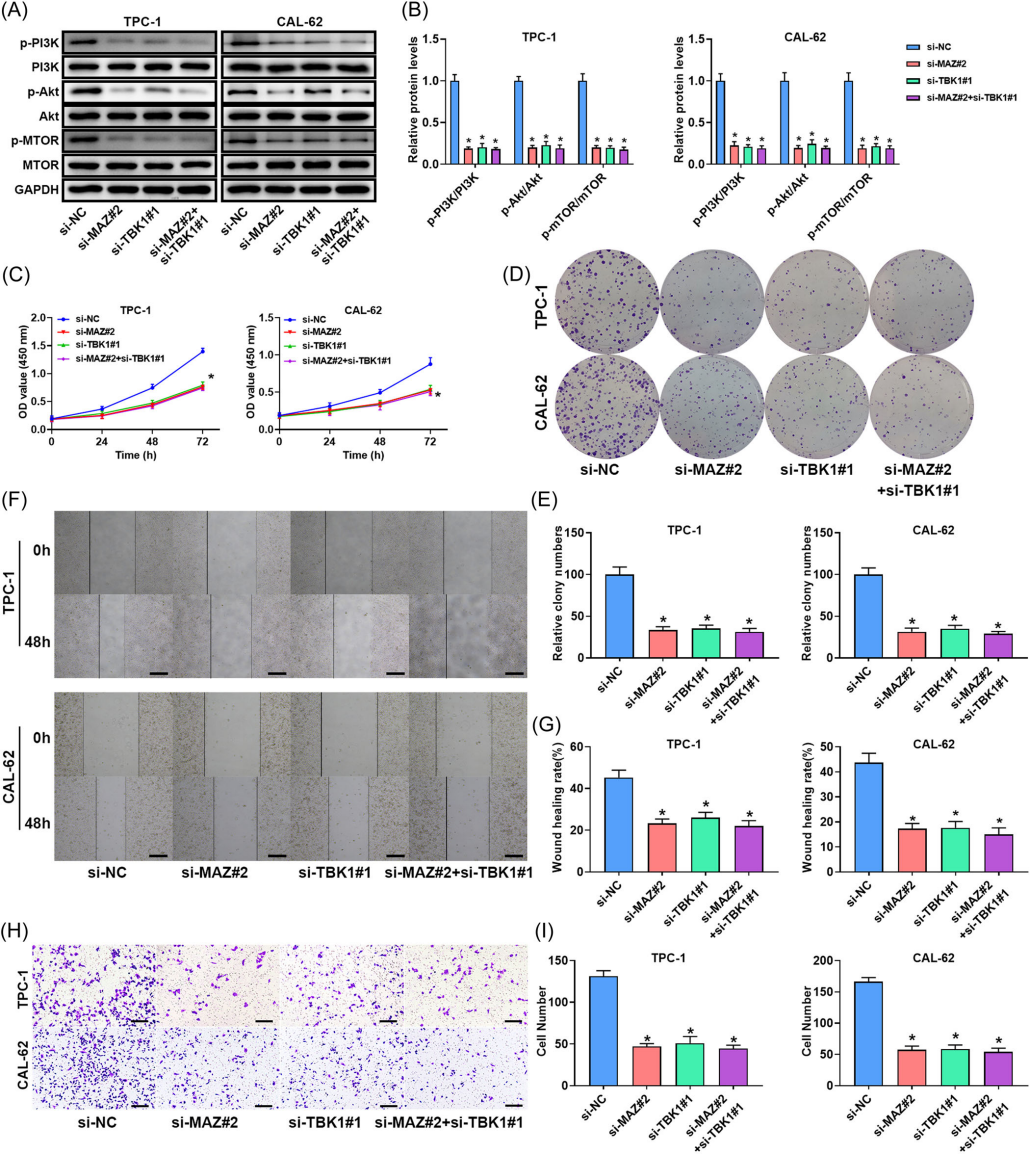
Effect of MAZ and TBK1 double knockout on thyroid cancer cells. (A, B) The expression of pathway‐related proteins in TPC‐1 and CAL‐62 cells. Band densities were determined using ImageJ Software and quantified as the ratio to GAPDH. Take the mean value for samples from the first group on each immunoblot, and then divide the value of all groups by that mean. The (C) cell viability, (D, E) proliferation, (F, G) migration, and (H, I) invasion of TPC‐1 and CAL‐62 cells. Scale bar = 100 μm. *p < .05. Akt, protein kinase B; GAPDH, glyceraldehyde 3‐phosphate dehydrogenase; MAZ, Myc‐associated zinc finger protein; mTOR, mammalian target of rapamycin protein; NC, negative control; PI3K, phosphatidylinositol‐3‐kinase; p‐Akt, phospho‐Akt; p‐mTOR, phospho‐mTOR; p‐PI3K, phospho‐PI3K; TBK1, TANK‐binding kinase 1.

To further check the regulatory effects of MAZ and TBK1, MAZ siRNA and TBK1 siRNA were cotransfected into thyroid cancer cells. Silencing MAZ and TBK1 alone significantly inhibited the PI3K/Akt/mTOR pathway in thyroid cancer cells, whereas cotransfection with MAZ siRNA and TBK1 siRNA did not strengthen the inhibitory effect of MAZ RNA or TBK1 siRNA on the PI3K/Akt/mTOR pathway (Figure 6A,B). Both MAZ and TBK1 silencing significantly decreased the viability and proliferation of thyroid cancer cells, whereas the inhibitory effect was not further enhanced by the cotransfection with MAZ siRNA and TBK1 siRNA (Figure 6C–E). MAZ and TBK1 silencing markedly inhibited the migration and invasion abilities, whereas the inhibitory effect was not further enhanced by cotransfection with MAZ siRNA and TBK1 siRNA (Figure 5F–I).

### 3.7 TBK1 silencing inhibited tumor growth in thyroid cancer

**Figure 7:**
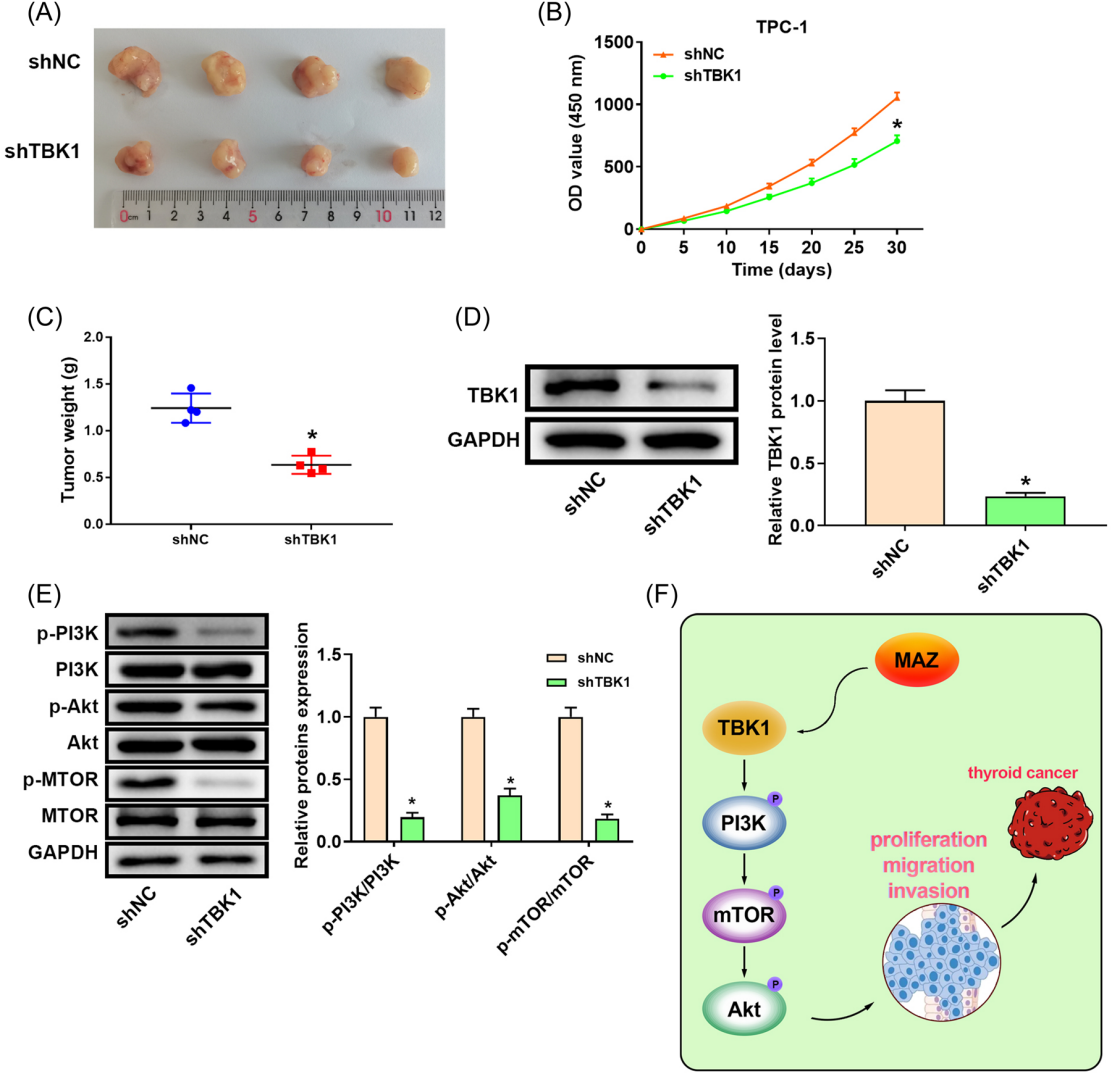
TBK1 silencing inhibited tumor growth of thyroid cancer. (A) Tumors excised from mice. The (B) tumor volume, (C) weight, (D) TBK1 expression, and (E) expression of pathway‐related proteins. Band densities were determined using ImageJ Software and quantified as the ratio to GAPDH. Take the mean value for samples from the first group on each immunoblot, and then divide the value of all groups by that mean. *p < .05. (F) A schematic model of TBK1 function in thyroid cancer. Akt, protein kinase B; GAPDH, glyceraldehyde 3‐phosphate dehydrogenase; MAZ, Myc‐associated zinc finger protein; mTOR, mammalian target of rapamycin protein; OD, optical density; PI3K, phosphatidylinositol‐3‐kinase; p‐Akt, phospho‐Akt; p‐mTOR, phospho‐mTOR; p‐PI3K, phospho‐PI3K; TBK1, TANK‐binding kinase 1.

To confirm the effect of TBK1 on tumor growth, TPC‐1 cells transfected with shTBK1 or shNC were subcutaneously injected into nude mice. Tumor growth was remarkably slower in mice injected with shTBK1 than in those injected with shNC (Figure 7A). The tumor in the shTBKi group had a lower weight and size than those in the sh‐NC group (Figure 7B,C). TBK1 expression was reduced in the shTBK1 group (Figure 6D). As shown in Figure 7E, TBK1 shRNA significantly inhibited the PI3K/Akt/mTOR pathway, manifested as a significant decrease of pathway‐associated proteins in the shTBK1 group. Briefly, TBK1 silencing repressed xenograft tumor growth in thyroid cancer cells.

## 4 DISCUSSION

Thyroid cancer leads to 41,071 deaths worldwide in 2018.^1^ TBK1 expression is upregulated in different types of human cancers.^26^, ^27^, ^28^ In this study, TBK1 expression was upregulated and a high level of TBK1 expression was related to the poor survival rate of patients with thyroid cancer. Moreover, TBK1 overexpression promoted proliferation, and invasion, whereas the opposite results were obtained in TBK1 silencing thyroid cancer cells. Notably, TBK1 activated the PI3K/Akt/mTOR pathway and was regulated by MAZ positively.

High TBK1 level was related to the recurrence pattern of metastasis in NSCLC compared to local recurrence.^29^ High TBK1 expression was correlated with poor prognosis and contributed to the vascular invasion of hepatocellular carcinoma.^26^ In this study, TBK1 expression was upregulated in thyroid cancer samples. Kaplan–Meier analysis indicated that thyroid cancer patients with high TBK1 levels showed low overall survival. Furthermore, the progression of thyroid cancer cells was markedly enhanced by TBK1 overexpression and inhibited by TBK1 silencing. The data suggested that TBK1 exerts a vital function in the promotion of thyroid tumorigenesis.

MAZ, a critical driver of inflammation in animal models,^30^ is abnormally expressed in cancers.^31^ Research by Triner et al. suggested that MAZ was overexpressed in human colon cancer.^32^ After MAZ knockout, cell proliferation was inhibited and the cell cycle was arrested in the G0/G1 phase in prostate cancer.^33^ Inhibition of MAZ can significantly inhibit cell migration of gastric cancer.^34^ Similarly, MAZ was overexpressed in thyroid cancer tissues. In addition, MAZ positively modulated TBK1. MAZ overexpression markedly increased TBK1 expression. MAZ silencing markedly inhibited the proliferation and migration of thyroid cancer cells and reversed the effect of TBK1 overexpression. Additionally, cotransfection with MAZ siRNA and TBK1 siRNA did not strengthen the inhibitory effect of MAZ siRNA or TBK1 siRNA alone. These results suggest that the proinflammatory effect of TBK1 on thyroid cancer cells can be inhibited by MAZ silencing.

PI3K/AKT/mTOR pathway plays a vital function in diseases and cancers.^35^, ^36^, ^37^ Activation of the PI3K/Akt pathway is involved in the survival and invasion of cancer cells.^38^, ^39^ Liu et al. demonstrated that the XIST/miR‐34a axis modulated tumor growth of thyroid cancer cells via the mesenchymal‐to‐epithelial transition–PI3K–Akt pathway.^40^ Activation of the PI3K/Akt pathway can partly abolish the inhibitory effect of ENST silencing on thyroid cancer cells.^41^ In our study, TBK1 activated the PI3K/Akt/mTOR signaling pathway. TBK1 overexpression promoted the levels of p‐PI3K/PI3K, p‐Akt/Akt, and p‐mTOR/mTOR, whereas MAZ silencing significantly inhibited the expression. Additionally, the activating effect of TBK1 on the PI3K/Akt/mTOR pathway could be partly reversed by MAZ silencing. These data suggested that the promoted effect of TBK1 on thyroid cancer was accomplished by activating the PI3K/Akt/mTOR pathway.

TBK1 promoted tumor progression of thyroid cancer cells by activating the PI3K/Akt/mTOR pathway. However, current research lacks the effect of TBK1 on the apoptosis of thyroid cancer cells. Whether TBK1 participates in thyroid cancer progression by regulating other pathways requires further investigation.

## AUTHOR CONTRIBUTIONS

**Qiuli Jiang**: Writing—original draft; writing—review and editing. **Yingying Guan**: Investigation. **Jingmei Zheng**: Investigation. **Huadong Lu**: Data curation.

## CONFLICT OF INTEREST STATEMENT

The authors declare no conflict of interest.

## ETHICS STATEMENT

The protocol of this research has been approved by the Ethics Committee of the Xiamen Branch, Zhongshan Hospital, Fudan University. All patients have signed written informed consent.

## Data Availability

The datasets used and analyzed during the current study are available from the corresponding author upon reasonable request.
